# Changes in choroidal thickness in advanced diabetic retinopathy treated with pan-retinal photocoagulation using a pattern scanning laser versus a conventional laser

**DOI:** 10.1186/s12886-020-01501-1

**Published:** 2020-06-12

**Authors:** Nari Park, In Gul Lee, Jee Taek Kim

**Affiliations:** 1 Department of Ophthalmology, College of Medicine, Chung-Ang University Hospital, 102 Heukseok-ro, Dongjak-gu, Seoul, 06974 South Korea; 2 Dangjin bright eye center, Dangjin, South Korea

**Keywords:** Choroidal thickness, Conventional laser, Diabetic retinopathy, Optical coherence tomography, Pan-retinal photocoagulation, Pattern scanning laser

## Abstract

**Background:**

To compare the effect of pan-retinal photocoagulation (PRP) using pattern scanning or conventional laser on subfoveal choroidal thickness (SFChT).

**Methods:**

Thirty-eight patients (64 eyes) with advanced diabetic retinopathy (DR) who underwent PRP using pattern scanning or conventional laser were included. Changes in SFChT were compared with baseline values at 1, 3, 6, and 12 months after PRP using swept-source optical coherence tomography.

**Results:**

The conventional laser group showed a statistically significant decrease in SFChT at 1, 3, 6, and 12 months after PRP (*P* < 0.001). SFChT was significantly decreased at 3 (*P* = 0.025), 6 (*P* = 0.004), and 12 (*P* < 0.001) months after treatment in the pattern laser group.

**Conclusion:**

Eyes with advanced DR showed a significant reduction in SFChT over 12 months regardless of the type of laser used; however, the reduction was sooner after conventional laser than after pattern laser.

## Background

Diabetic retinopathy (DR) is a common complication of diabetes and a leading cause of vision loss in working-aged populations in both advanced and developing countries [1]. Pan-retinal photocoagulation (PRP) is considered the gold standard treatment for severe non-proliferative diabetic retinopathy (NPDR) and proliferative retinopathy (PDR) according to the Early Treatment Diabetic Retinopathy Study (ETDRS) [2]. Traditionally, PRP has been performed using various wavelengths, including argon green (514 nm) or diode (810 nm) lasers. However, these lasers have recently been replaced by a pattern scanning laser system that uses a frequency-doubled 532 nm wavelength Nd:YAG laser to avoid patient discomfort and fatigue. Furthermore, the reduced pulse duration required when using the pattern scanning laser may be less painful because of decreased thermal diffusion into the choroid [3].

Several studies have shown that SFChT is affected by treatment, including PRP and intravitreal injections [4–9]. However, there have been no studies comparing the effect of conventional laser and pattern scanning laser systems on choroidal thickness. It has been reported that PRP performed with the pattern scanning laser is less effective than that performed with a traditional laser for regression of retinal neovascularization in eyes with high-risk PDR [10]. The purpose of this study was to compare the effect of PRP on choroidal thickness using the conventional and pattern scanning lasers.

## Methods

### Subjects

This retrospective interventional comparative study was approved by the institutional review board committee of Chung-Ang University Hospital, Seoul, South Korea, and adhered to the tenets of the Declaration of Helsinki. The medical records of consecutive patients who underwent PRP for PDR or severe NPDR at the Chung-Ang University Hospital from September 1, 2015 to July 31, 2017 and who were followed up for at least 12 months were retrospectively reviewed. A signed informed consent was obtained from all patients before each PRP session. The exclusion criteria included systemic disease other than diabetes, prior retinal surgery or photocoagulation, any history of eye disease, retinal disease or choroidal disease, a history of ocular trauma, and refractive error ≥ ± 3.0 diopters.

Furthermore, patients who received anti-vascular endothelial growth factor (VEGF) injection before the baseline date and during the follow-up period were excluded, as were patients who underwent cataract surgery within 6 months before the baseline date and during follow-up.

Eyes with low-quality OCT images (an image quality index < 90) because of media opacities such as vitreous hemorrhage or cataract were additionally excluded. However, eyes with good-quality OCT images and localized vitreous hemorrhage or peripheral cortical lens opacity were not excluded.

All patients underwent a comprehensive ophthalmic examination, an fluorescein angiography, and swept-source OCT (DRI Triton OCT, Topcon, Tokyo, Japan). The severity of DR, central retinal thickness (CRT), and SFChT were determined by previously described methods [11].

### Pan-retinal photocoagulation

PRP was done by a single retinal specialist (JTK) with a 532 nm frequency-doubled neodymium-doped yttrium aluminum garnet (Nd-YAG) solid-state pattern scan laser (Valon pattern laser, Dual Laser Ltd. Oy, Finland) or a 532 nm solid-state green diode laser (OcuLight GLx laser, Iridex Corp. Mountain View, CA, USA). The patients were classified into a pattern laser group or a conventional laser group as appropriate. Patients underwent PRP with a conventional diode laser before January 2017 and PRP with a pattern laser from January 2017 onwards after acquisition of the new laser. PRP was placed from the superior and inferior vascular arcades to the peripheral retina and performed in all eyes in both groups in two sessions one week apart.

For pattern laser, a 5 × 5 multispot array with a 200 μm spot size, a 20 ms pulse duration, and a 1.5 width spot spacing was used. The laser power was adjusted from 200 mW until a gray-white opacity was achieved. For conventional laser, the laser was performed by previously described [11].

### Statistical analysis

The data are expressed as the mean ± standard deviation. The CRT, SFChT, reduction in SFChT (ΔSFChT), and rate of reduction in SFChT (%ΔSFChT) were analyzed during follow-up. Statistical analyses were done using SPSS version 23.0 software (IBM Corp., Armonk, NY, USA) with the independent *t*-test, paired *t*-test, and chi-square test as appropriate. A *P*-value < 0.05 was considered statistically significant.

## Results

### Baseline characteristics

Sixty-four eyes (38 patients) with severe NPDR (*n* = 27) or PDR (*n* = 37) were included in this study. There were 37 eyes in the conventional laser group and 27 eyes in the pattern laser group. The mean patient age was 55.2 ± 11.7 (range 34–71) years, and the mean HbA_1c_ value was 8.0% ± 1.8%, the mean duration of diabetes was 13.2 ± 5.8 (range 7–22) years. The mean BCVA was 0.24 ± 0.25 logMAR, the mean spherical equivalent was − 0.73 ± 1.8, and the mean intraocular pressure was 15.2 ± 3.3 mmHg. There was no significant difference in age, sex, refractive error, HbA_1c_, BCVA, or mean duration of diabetes between the two study groups (Table 1).

**Table 1:** Demographic and clinical characteristics of patients included in the study

	Total (64 eyes)	Conventional laser group (37 eyes)	Pattern laser group (27 eyes)	*P*-value
Age (years)	55.2 ± 11.7	55.3 ± 11.3	55.1 ± 12.7	0.936^a^
Sex M/F (no.)	37/27	22/15	15/12	0.755^b^
HbA_1c_ (%)	8.0 ± 1.8	8.3 ± 2.2	7.8 ± 1.5	0.33^a^
Duration of DM (years)	13.2 ± 5.8	13.1 ± 4.8	13.3 ± 6.7	0.56^a^
BCVA (logMAR)	0.24 ± 0.25	0.27 ± 0.29	0.21 ± 0.2	0.486^a^
IOP (mmHg)	15.2 ± 3.3	14.2 ± 3.6	16.2 ± 2.6	0.068^a^
SE (diopter)	−0.73 ± 1.8	−0.63 ± 1.8	−0.75 ± 1.8	0.944^a^
PDR/Severe NPDR (no.)	37/27	22/15	15/12	0.477^b^
Baseline CRT (μm)	266.2 ± 87.4	284.1 ± 104.7	250.3 ± 67.4	0.166^a^

*BCVA* best-corrected visual acuity, *CRT* central retinal thickness, *DM* diabetes mellitus, *HbA*_*1c*_ glycated hemoglobin, *IOP* intraocular pressure, *NPDR* non-proliferative retinopathy, *PDR* proliferative retinopathy, *SE* spherical equivalent

^a^Independent t-test; ^b^chi-square test

PRP was performed using a mean power of 318.5 ± 285.7 (range 210–500) mW in the conventional laser group and a mean power of 355.9 ± 111.5 (range 240–540) mW in the pattern laser group (*P* = 0.061). The total number of photocoagulation burns was 1512.8 ± 246.8 in the conventional laser group and 3216.8 ± 287.3 in the pattern laser group (*P* < 0.001).

There was no correlation between the mean baseline SFChT and age (*P* = 0.138), HbA_1c_ (*P* = 0.237), BCVA (*P* = 0.747), or spherical equivalent (*P* = 0.795). The inter-observer reproducibility of the SFChT measurement ranged from 0.986 to 0.990. The baseline CRT was similar in both groups and there was no statistically significant difference in the changes in CRT following PRP between the study groups.

### Mean subfoveal choroidal thickness in the conventional and pattern laser groups

**Fig. 1:**
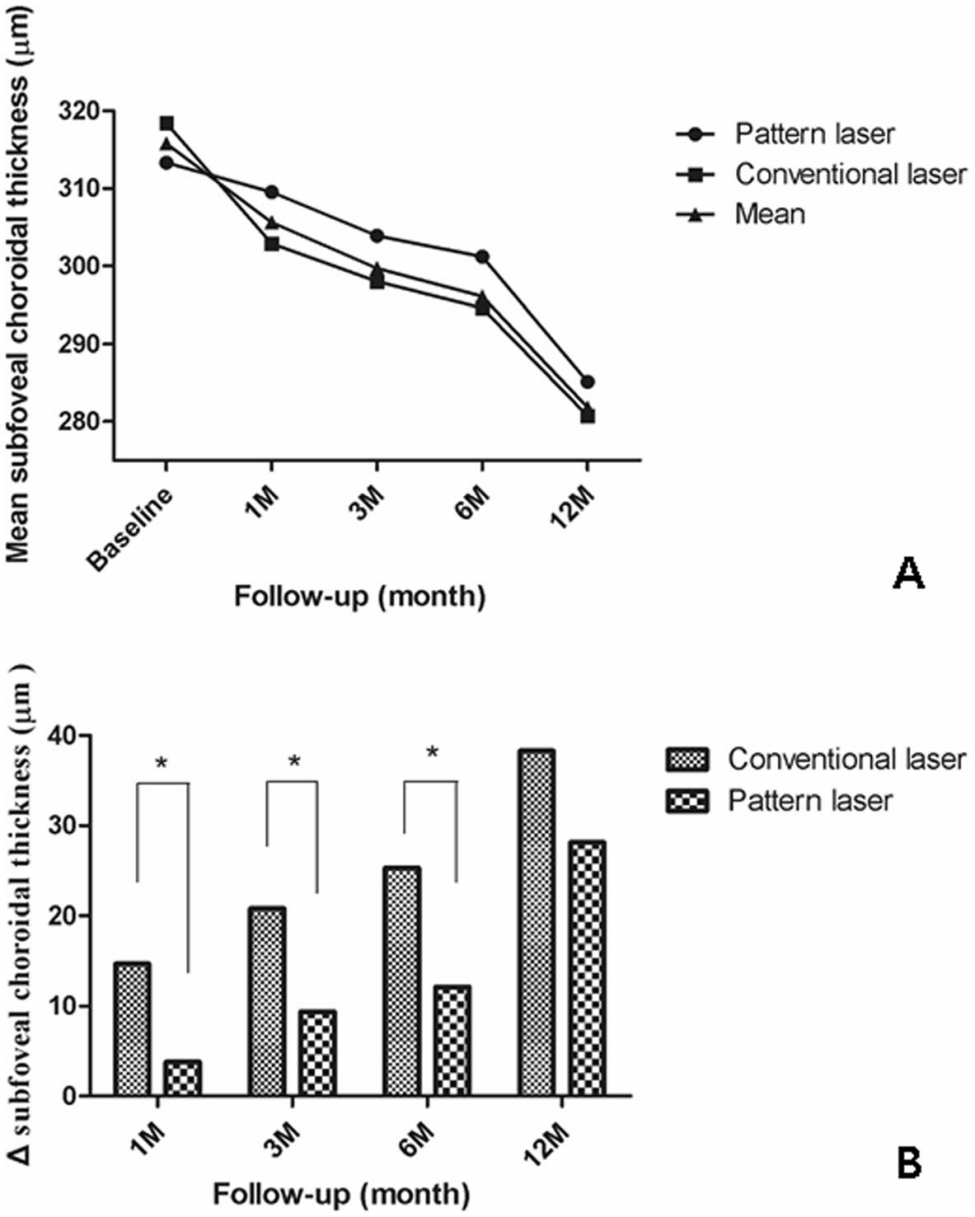
Comparison of the changes and reduction in SFChT after pan-retinal photocoagulation between the conventional laser and pattern laser groups. **a** Comparison of changes in mean SFChT between the two groups. The mean SFChT was significantly decreased during follow-up. **b** Comparisons of the reduction in SFChT between the two groups. The reduction was more prominent in the conventional laser group than in the pattern group, especially in the early period after pan-retinal photocoagulation. SFChT, mean subfoveal choroidal thickness; ΔSFChT, reduction of SFChT = SFChT at baseline – SFChT at time point. *P < 0.05. SFChT, subfoveal choroidal thickness

The mean SFChT in the conventional laser group was 318.4 ± 58.3 μm at baseline and decreased significantly (*P* < 0.001 vs baseline) at 1, 3, 6 and 12 months, respectively (Fig. 1a).

The mean SFChT in the pattern laser group was 313.3 ± 91.9 μm at baseline. There were no significant changes in the mean SFChT between baseline and 1 month (309.5 ± 93.4 μm, *P* = 0.404 vs baseline) after PRP. The mean SFChT were significantly decreased to 309.5 ± 93.4 μm at 3 months (*P* = 0.404 vs baseline), 301.2 ± 89.6 μm at 6 months (*P* = 0.004 vs baseline), and 285.1 ± 87.6 μm at 12 months (*P* < 0.000 vs baseline; Fig. 1a).

ΔSFChT was significantly greater in the conventional laser group than in the pattern laser group at 1 month (14.7 ± 17.3 μm vs 3.8 ± 21.9 μm, *P* = 0.03), 3 months (20.8 ± 18.6 μm vs 9.4 ± 19.1 μm, *P* = 0.027), and 6 months (25.3 ± 21.2 μm vs 12.1 ± 18.5 μm, *P* = 0.018) after PRP. There was no statistically significant difference at 12 months (38.3 ± 19.2 μm vs 28.1 ± 22.8 μm, *P* = 0.075) after PRP (Fig. 1b, Table 2).

**Table 2:** Comparison of mean subfoveal choroidal thickness between the two study groups during follow-up after pan-retinal photocoagulation

		Changes in mean SFChT according to laser used			Changes in mean SFChT according to severity of DR		
	Total (*n* = 64)	Pattern laser group (*n* = 27)	Conventional laser group (*n* = 37)	^a^*P-*value	Severe NPDR group (*n* = 27)	PDR group (*n* = 37)	^a^*P*-value
SFChT Baseline	315.8 ± 73.3	313.3 ± 91.9	318.4 ± 58.3	0.798	309.1 ± 74.3	319.4 ± 73.5	0.617
SFChT 1 M	305.6 ± 71.6	309.5 ± 93.4	302.9 ± 52.7	0.742	297.4 ± 72.5	310.1 ± 71.8	0.527
SFChT 3 M	299.7 ± 69.6	303.9 ± 89.6	298.0 ± 52.3	0.758	295.4 ± 72.7	302.1 ± 68.8	0.73
SFChT 6 M	296.1 ± 72.6	301.2 ± 89.6	294.6 ± 57.9	0.74	290.2 ± 74.4	296.0 ± 72.6	0.993
SFChT 12 M	281.8 ± 69.4	285.1 ± 87.6	280.7 ± 53.9	0.819	284.2 ± 71.6	280.4 ± 69.1	0.847
Δ SFChT 1 M	10.1 ± 20.1	3.8 ± 21.9	14.7 ± 17.3	0.03	11.7 ± 15.5	9.3 ± 22.3	0.666
Δ SFChT 3 M	16.1 ± 19.5	9.4 ± 19.1	20.8 ± 18.6	0.027	13.7 ± 16.9	17.3 ± 20.9	0.519
Δ SFChT 6 M	19.7 ± 21.0	12.1 ± 18.5	25.3 ± 21.2	0.018	12.9 ± 16.2	23.4 ± 22.5	0.072
Δ SFChT 12 M	34.0 ± 21.2	28.1 ± 22.8	38.3 ± 19.2	0.075	24.8 ± 13.7	38.9 ± 22.9	0.006
%Δ SFChT 1 M	2.9 ± 6.2	1.1 ± 7.2	4.5 ± 5.1	0.52	3.7 ± 4.8	2.6 ± 6.9	0.525
%Δ SFChT 3 M	4.8 ± 6.1	2.7 ± 6.4	6.3 ± 5.5	0.03	4.4 ± 5.3	5.0 ± 6.6	0.701
%Δ SFChT 6 M	6.2 ± 6.8	3.7 ± 6.4	7.8 ± 6.7	0.02	4.2 ± 5.6	7.2 ± 7.3	0.121
%Δ SFChT 12 M	10.7 ± 6.6	8.9 ± 7.2	11.9 ± 5.9	0.087	8.1 ± 4.2	12.2 ± 7.3	0.01

*NPDR* non-proliferative retinopathy, *PDR* proliferative retinopathy, *SFChT* subfoveal choroidal thickness, *ΔSFChT* reduction of *SFChT* SFChT at baseline – SFChT at time point, *%ΔSFChT* percentage of reduction in SFChT = 100 × (SFChT at baseline – SFChT at time point)/SFChT at baseline

^a^Independent *t*-test

The %ΔSFChT was also greater in the conventional laser group than in the pattern laser group at 1 month (4.5% ± 5.1% vs 1.1% ± 7.2%, *P* = 0.52), 3 months (6.3% ± 5.5% vs 2.7% ± 6.4%, *P* = 0.03), 6 months (7.8% ± 6.7% vs 3.7% ± 6.4%, *P* = 0.02), and 12 months (11.9% ± 5.9% vs 8.9% ± 7.2%, *P* = 0.087; Table 2).

### Changes in subfoveal choroidal thickness between the severe NPDR and PDR groups

**Fig. 2:**
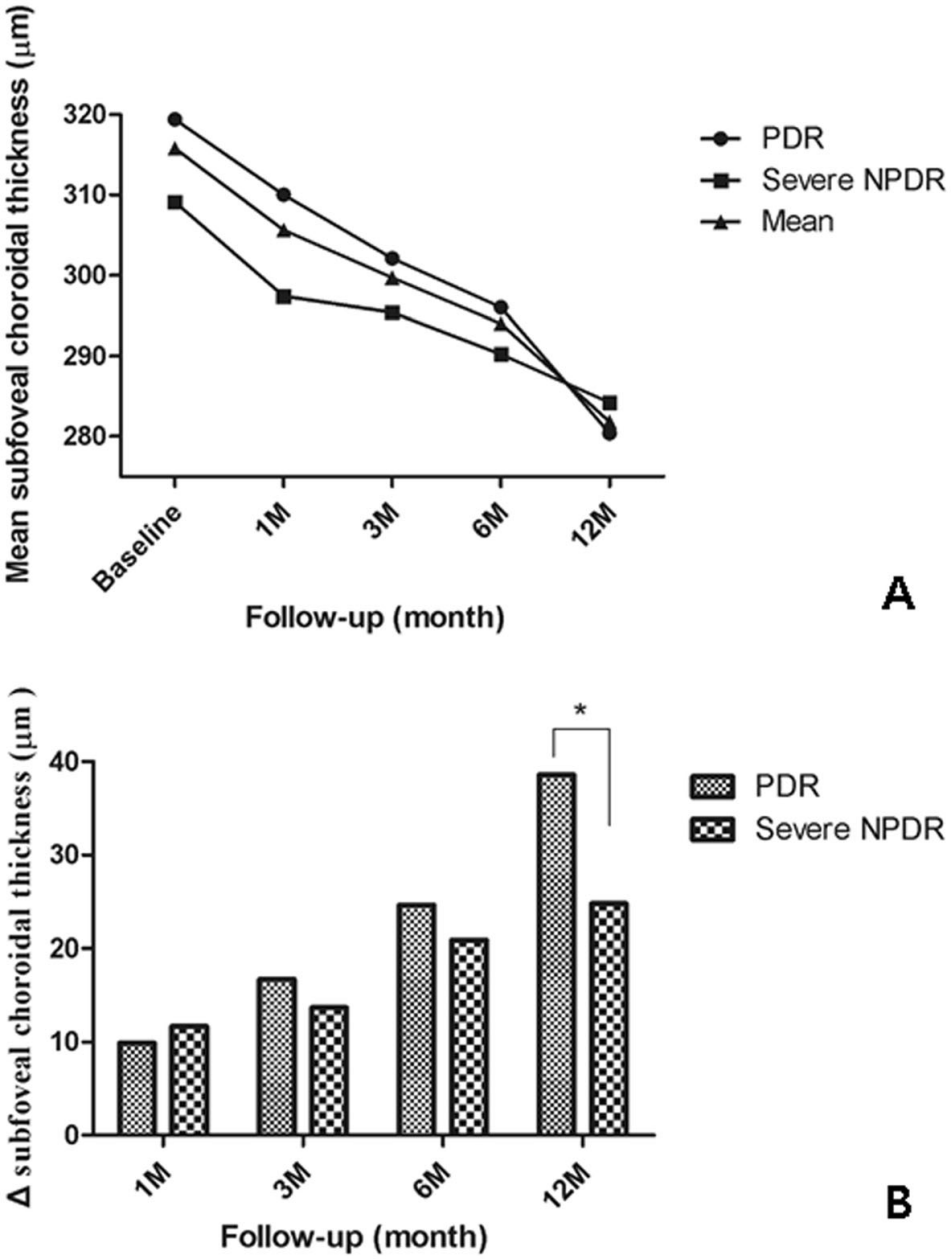
Comparisons of the changes and reduction in mean subfoveal choroidal thickness (SFChT) after pan-retinal photocoagulation between the PDR and severe NPDR groups. **a** Comparison of changes in mean SFChT between the two groups. **b** Comparison of the reduction in SFChT between the two groups. The PDR group showed a more prominent reduction than the severe NPDR group at 12 months after pan-retinal photocoagulation. ΔSFChT, reduction of SFChT = SFChT at baseline – SFChT at time point. PDR, proliferative retinopathy; NPDR, non-proliferative retinopathy *P = 0.006

Thirty-seven of the 64 eyes (38 patients) had PDR and 27 had severe NPDR. The mean SFChT in the PDR group was 319.4 ± 73.5 μm at baseline and decreased significantly through the follow-up period after PRP. The mean SFChT in the severe NPDR group was 309.1 ± 74.3 μm at baseline and also decreased significantly at every time point after PRP (Fig. 2a).

The ΔSFChT values in the PDR and severe NPDR groups were 9.9 ± 22.3 μm and 11.7 ± 15.5 μm, respectively, at 1 month, 16.7 ± 20.9 μm and 13.7 ± 16.8 at 3 months, and 24.6 ± 22.5 and 20.9 ± 16.2 at 6 months. There was no statistically significant difference in the amount of reduction in ΔSFChT between the two groups. However, the ΔSFChT was significantly greater in the PDR group than in the severe NPDR group at 12 months after PRP (38.6 ± 22.9 μm vs 24.8 ± 13.7, *P* = 0.006; Fig. 2b; Table 2).

The %ΔSFChT values in the PDR and severe NPDR groups were 2.7% ± 6.9 and 3.6% ± 4.8%, respectively, at 1 month, 4.9% ± 6.5 and 4.4% ± 5.2% at 3 months, and 7.6% ± 7.3 and 7.3% ± 5.6% at 6 months. There was no statistically significant difference in the amount of change in %ΔSFChT between the two groups. However, the %ΔSFChT was significantly greater in the PDR group than in the severe NPDR group at 12 months after PRP (12.1% ± 7.3% vs 8.1% ± 4.2%, *P* = 0.01; Table 2).

## Discussion

In this study, we compared the changes in SFChT in eyes with advanced DR after PRP between a conventional laser group and a pattern laser group. There was a significant decrease in SFChT after PRP compared with the baseline in both groups of eyes during the follow-up period. The reduction in SFChT was more prominent in the conventional laser group than in the pattern laser group at an early stage following PRP.

Blumenkranz et al. have developed a pattern scanning laser with a shorter pulse duration of 10–20 ms that uses a Nd:YAG laser with a 532 nm wavelength [3]. They reduced the pulse duration of the laser to decrease patient discomfort and to reduce the amount of time taken to perform PRP. The shorter pulse duration requires less pulse energy. The laser scars produced by the shorter pulses are smaller than those produced by longer pulses [12]. Nagpal et al. reported that the average scar size created by the conventional laser was significantly greater than that created by the pattern scanning laser (430 μm vs 310 μm) at 3 months after PRP with the same 200 μm spot size [13]. Photocoagulation scars created using a conventional laser with a pulse duration of 100–200 ms tend to enlarge progressively because of heat diffusion [12]. Therefore, it seems that the difference in the changes in SFChT between the two groups after PRP in this study was related to differences in the expansion of the laser burn scar. Moreover, the laser fluence created by the pattern scanning laser was significantly less than that created by the conventional laser (40.33 J/cm^2^ vs 191 J/cm^2^) [13].

Several studies have shown that the SFChT decreases after PRP and that PRP-treated eyes have a smaller SFChT than naïve eyes [4, 8, 9, 14–16]. Zhang et al. found that the SFChT decreased significantly 3 months after PRP [8], as did Okamoto et al. [9], while Ohara et al. found that SFChT decreased significantly 6 months after PRP [16] and Kang et al. reported a significant reduction in the SFChT 12 months after PRP [14]. .The present study also showed that the SFChT was significantly reduced over 12 months after PRP. This finding is consistent with the earlier research with regards to a long-lasting PRP effect [14]. Previously, Maeshima et al. described progressive enlargement of the laser scar over several years [17]. Moreover, they reported that the mean annual laser scar expansion rates were 7–12.7%. We suggest that the long-lasting effect on SFChT over 12 months is associated with expansion of the laser burn scar.

Despite the consistent finding of a decrease in the SFChT during long-term observation, reports on the more short-term studies have been conflicting but suggest a significant increase in SFChT at 1–12 weeks after PRP [18–20]. It is thought that the inconsistent early changes in SFChT after PRP might be caused by release of inflammatory cytokines in response to laser photocoagulation. Four eyes in our pattern laser group and two eyes in our conventional laser group showed thickening of the SFChT by more than 5% ΔSFChT. It seems that eyes that received higher laser energies are more likely to have thickened SFChT in the early phase after PRP than eyes that received lower laser energies. Thus, we think that a subgroup analysis according to different laser energies or glycemic control status would be interesting. However, the number of eyes was too small to obtain statistically significant results.

We also compared the changes in SFChT after PRP between the PDR and severe NPDR groups. The two groups showed a similar tendency for a decrease in SFChT during the study period. However, the reduction in SFChT was more prominent in the PDR group than in the severe NPDR group at 12 months following PRP. The reduction in SFChT after PRP may be caused by a decrease in the level of vascular endothelial growth factor (VEGF) secreted from the non-perfusion area in response to ablation of an ischemic retina and choriocapillaris. There have been reports of a significant reduction in SFChT in eyes treated with intravitreal injections of anti-VEGF or triamcinolone acetonide as well as PRP [21–23]. These findings also suggest that the choroid tissue is sensitive to the amount of VEGF or cytokines released from the retinal tissue.

Essentially, we have attempted to analyze the correlation between the change in DR stage and SFChT after PRP. Before PRP, the DR stage was usually evaluated by FAG. However, FAG is not regularly performed to analyze the regression of new vessels in eyes with proliferative DR and rarely performed in eyes with severe NPDR after PRP. Therefore, a prospective study will be needed to analyze the correlation between the regression of DR stage and the changes in SFChT after PRP.

This study has several limitations. First, is its small sample size. The second is its retrospective rather than controlled prospective design. Third, recurrence or persistence of new vessels in eyes with PDR after PRP was not considered. Fourth, eyes with severe or high-risk PDR were not included in this study, because most received additional PRP or vitrectomy due to persistent new vessels or vitreous hemorrhage. Fifth, diurnal variation was not considered. Sixth, the effect of intravitreal anti-VEGF injection was not considered in eyes with DME. However, despite these limitations, this is the first study to compare the effect of pattern scanning laser with that of conventional laser on SFChT after PRP. Further investigations with a prospective design are needed to confirm the findings of the present study.

## Conclusion

SFChT is reduced significantly after PRP. However, the effect of conventional laser on the SFChT is greater than that of pattern scanning laser, especially in the early stages after PRP.

## Data Availability

The datasets used and/or analyzed in this study are available from the corresponding author on reasonable request.

## Abbreviations

BCVA: best-corrected visual acuity
CRT: central retinal thickness
DR: diabetic retinopathy
ETDRS: Early Treatment Diabetic Retinopathy Study
Nd-YAG: neodymium-doped yttrium aluminum garnet
NPDR: non-proliferative diabetic retinopathy
PDR: proliferative retinopathy
PRP: pan-retinal photocoagulation
SFChT: subfoveal choroidal thickness
ΔSFChT: reduction in SFChT
%ΔSFChT: rate of reduction in SFChT
